# A systematic review of qualitative research on barriers and facilitators to exclusive breastfeeding practice in sub-Saharan African countries

**DOI:** 10.1186/s13006-021-00380-6

**Published:** 2021-06-05

**Authors:** Izuchukwu Loveth Ejie, George Uchenna Eleje, Moriam Taiwo Chibuzor, Maureen Ugonwa Anetoh, Ifeoma Jovita Nduka, Ifeoma Blessing Umeh, Brian Onyebuchi Ogbonna, Obinna Ikechukwu Ekwunife

**Affiliations:** 1 Department of Clinical Pharmacy and Pharmacy Management, Faculty of Pharmaceutical Sciences, Nnamdi Azikiwe University, Awka, Nigeria; 2 Research Group for Evidence-Based Health Care, Nnamdi Azikiwe University, Awka, Nigeria; 3 Department of Obstetrics and Gynaecology, Nnamdi Azikiwe University Teaching Hospital, Nnewi, Nigeria; 4 Effective Care Research Unit, Department of Obstetrics and Gynaecology, Nnamdi Azikiwe University, Awka, Nigeria; 5 Calabar Institute of Tropical Diseases Research and Prevention, University of Calabar Teaching Hospital, Moore Road, Calabar, Nigeria

**Keywords:** Barriers, Breastfeeding, Exclusive breastfeeding, Facilitators, Breastfeeding mothers, Qualitative studies, Sub-Saharan Africa

## Abstract

**Background:**

Studies reporting factors associated with exclusive breastfeeding are mostly quantitative. No study has performed a systematic qualitative summary to document the recurring constraints and facilitators to exclusive breastfeeding in sub-Saharan African countries from breastfeeding mothers’ perspective. This study systematically reviews the literature reporting barriers and facilitators to exclusive breastfeeding from the breastfeeding mothers’ perspective in sub-Saharan Africa to develop an educational intervention to optimize exclusive breastfeeding.

**Methods:**

A systematic literature review of qualitative studies such as phenomenological studies, followed by a risk of bias and methodological assessment of the included studies’ quality using the Critical Appraisal Skills Programme (CASP) tool was conducted. MEDLINE and Google Scholar were searched from January 1990 to October 2019 to retrieve studies of breastfeeding mothers who had infants aged between 0 and 12 months. Two authors independently carried out the review process and resolved disagreements through consensus. We analyzed the data thematically.

**Results:**

After reviewing 92 studies, 20 studies involving 836 participants from 11 countries were eligible. Of the 72 studies excluded, 39 were not conducted in sub-Saharan Africa, and 33 included other participants such as fathers. Three themes emerged as barriers to exclusive breastfeeding (EBF) and five additional themes were identified with facilitators of maternal-infant factors being the most significant in both cases. Maternal employment and knowledge of the benefits of EBF were the most common maternal-infant factors that served as a barrier and a facilitator, respectively. The study’s limitations were that the review involved only primary research among breastfeeding mothers living in sub-Saharan Africa and excluded studies not available in the English language. The information synthesized from this review could be used to develop communication strategies employed during individual and group patient education in the hospitals to improve breastfeeding mothers’ understanding, acceptance, and practice of exclusive breastfeeding. This review was prospectively registered with the International Prospective Register of Systematic Reviews (PROSPERO), registration number CRD42020133761.

**Conclusions:**

This review found that maternal-infant factors have the most significant influence over the practice of exclusive breastfeeding. Therefore, interventions targeted towards maternal-infant factors will improve and optimize exclusive breastfeeding significantly and, ultimately, improve maternal-child health outcomes.

**Supplementary Information:**

The online version contains supplementary material available at 10.1186/s13006-021-00380-6.

## Background

Exclusive breastfeeding (EBF) gives infants only breast milk from birth up to 6 months of age without other liquids or solids, not even water, except oral rehydration solution or drops/syrups of vitamins, minerals, or medicines [1]. It is one of the critical evidence-based interventions for child survival [2]. In sub-Saharan Africa, almost all infants are breastfed, while EBF is less common [3]. The region still faces high infant and child mortality rates [3].

Despite the benefits and efforts to promote breastfeeding, EBF is practiced sub-optimally in many low-to-middle income countries. Exclusive breastfeeding coverage of 90% could reduce child death rates in low-income countries by 11.6% [2, 4]. Only 35% of infants are exclusively breastfed worldwide [5]. In sub-Saharan Africa, a region with high infant and child mortality rates, only 33% of infants are exclusively breastfed [5, 6].

The Baby-friendly Hospital Initiative launched by WHO and UNICEF in 1991, implemented practices that protect, promote and support breastfeeding [7]. Several studies have shown that different factors including social class, level of education, maternal age, lack of parental support, living with a partner, employment status, parity, place of delivery, smoking during pregnancy and the presence of Baby-friendly Hospital Initiative policies are associated with EBF in high-income countries [8, 9]. Similarly, in low-income countries sociodemographic factors like maternal age, education, employment, residency, cultural and religious practices, in addition to living arrangements, antenatal care practices, home delivery and professional assistance at birth have been shown to be associated with suboptimal breastfeeding practices [10–15]. Understanding these factors has been considered necessary when supporting mothers and has been reviewed in several studies [16–20].

### Why it is essential to do this review

The rationale for this review is that while studies have reported factors associated with exclusive breastfeeding there has been only one review in the last 5 years documenting the constraints and facilitators to EBF in low-income countries, but it did not focus on sub-Saharan African countries [21]. This systematic review did not assess the methodological quality of the included studies and did not include HIV positive mothers. Aside from serving as an update, the current review assesses the methodological quality of included studies and those involving HIV positive mothers. The information synthesized from this review could be used to develop communication strategies employed during individual and group patient education in the hospitals (e.g., antenatal, or postnatal visits) to improve breastfeeding mothers’ understanding, acceptance, and practice of exclusive breastfeeding. The review participants are breastfeeding mothers with infants aged between 0 and 12 months, the intervention is exclusive breastfeeding, a comparator (does not apply), and the outcomes are barriers and facilitators to exclusive breastfeeding and the focus is qualitative study designs. The aim of the study is to systematically review the current literature on recurring barriers and facilitators of exclusive breastfeeding.

## Methods

This systematic review was registered prospectively with the International Prospective Register of Systematic Reviews (PROSPERO) with registration number CRD42020133761.

### Search strategy

We searched the electronic databases of MEDLINE (via PubMed) and Google Scholar for entries between January 1990 and October 2019, using the Cochrane Qualitative Research Methods Group [22]. The search strategy incorporated the following terms: ‘exclusive breastfeeding’, ‘breastfeeding’, ‘breast milk’, ‘sub-Saharan Africa’ and their associated synonyms. An information specialist with knowledge of systematic reviews developed the search strategy (Additional file 1) and searched PubMed. Additionally, we performed forward citation searching of relevant articles on Google Scholar and conducted a search for related articles on PubMed using the similar items feature.

### Study population

The study population was defined as breastfeeding mothers with infants aged between 0 and 12 months.

### Study selection

Two authors independently screened the search outputs (title and abstracts) for potentially eligible studies using the predefined eligibility criteria. We obtained full articles of all citations that were likely to meet the predefined selection criteria. We resolved disagreements on studies for inclusion through a discussion between the review authors and consensus to minimize bias. Where data were incomplete, including those in conference abstracts, the paper’s authors were contacted by email and if we received no response after 2 weeks, an additional email was sent. We evaluated all identified articles following a standardized predefined format. We selected all studies written in the English language.

### Study inclusion criteria

#### Types of studies

We included studies with a qualitative study design such as mixed methods, ethnographic designs, case studies, grounded theory, phenomenological studies or those that used qualitative methods for data collection such as interviews (individual and focus group), observation and other methods such as thematic analysis for the data analysis.

#### Types of participants

We included studies conducted in sub-Saharan Africa which focused on breastfeeding mothers with infants aged between 0 and 12 months.

#### Intervention

Exclusive breastfeeding.

#### Comparison

This does not apply.

#### Outcomes

Barriers and facilitators to EBF identified in qualitative study designs.

Studies were included if they involved breastfeeding mothers with infants aged between 0 and 12 months and employed qualitative study designs such as mixed methods, ethnographic designs, case studies, grounded theory, phenomenological studies, or they used qualitative methods for data collection such as interviews (individual and focus group), observation and qualitative methods such as thematic analysis.

### Study exclusion criteria

Studies were excluded if they utilized survey data or statistical reporting of results, or collected data using qualitative methods but analyzed the data quantitatively. Studies involving populations outside sub-Saharan African countries were excluded. We also excluded studies with an adjudged total score quality of less than six.

### Data extraction, quality appraisal and risk of bias assessment

Two authors independently screened the search outputs for potentially eligible studies using the predefined eligibility criteria. We resolved disagreements through discussion between the authors and consensus to minimize bias. The search results were presented in the form of a flow diagram as recommended by the Preferred Reporting Items for Systematic Reviews and Meta-analysis (PRISMA) [23] (Additional file 2). We extracted data from the 20 articles that met the inclusion criteria using a pre-tested data extraction tool (Additional file 3). For each study, we extracted the following data: the bibliographic information (author, year, country), study aims, study designs, participants, results (EBF barriers or facilitators, or both barriers and facilitators) and conclusions (Additional file 4). For quality assessment, we adopted the CASP tool for qualitative studies [24] to appraise the included studies’ methods, credibility and relevance. The tool was also used to assess the risk of bias of the included studies. The checklist consisted 10 questions to appraise the quality of qualitative research; responses scored as either Yes (1), No (0) or Unclear (0.5). We classified studies as high, moderate or low-quality if they had a total score of 9–10, 7.5–9, or 6–7.5, respectively. Studies with less than six scores were excluded (See Additional file 5 for details of the questions). At the second level of appraisal (risk of bias assessment), methodological limitations of emergent findings were considered and categorized as minor (low risk of bias), moderate (moderate risk of bias) or major (high risk of bias) (Additional files 6 & 7). These categorizations were based on the presence or absence of a description of key elements of the methodology (guided by the assessment in Additional file 4) in the document(s) which support the finding, including the following: the approach used to recruit or sample participants, how the potential for researcher bias was addressed and how the analysis was done. Minor methodological limitations were those with one element missing, moderate limitations were those with two elements missing and major limitations were those with three or more missing. Full details of which elements were missing from each document are available in Additional file 5. A thematic data synthesis was used to combine results. This study was limited to a qualitative systematic review and did not include a meta-analysis.

### Data synthesis

The extracted data were analyzed using thematic analysis techniques to identify themes arising from the data and facilitate a higher order of an abstraction and theory development. The thematic analysis and meta-synthesis processes outlined by Thomas and Harden (2008) were used to enhance transparency during the review process [25]. The data analysis was primarily undertaken by one reviewer; the findings discussed in team meetings to ensure they appropriately reflected the original data.

## Results

The search process returned a total of 2717 records from the electronic databases and 3956 records from forward citation searches of relevant articles on Google scholar. We removed any duplicates using EndNote software. Of the remaining 5838 records screened by title and abstract, 92 records were eligible. After reading the full text of the 92 studies, we excluded 72 studies that did not meet the eligibility criteria.

Twenty studies involving 800 and 36 participants from 11 countries were included. All 20 studies were included after the CASP tool was used to assess the methodological quality, with no study with a score of less than six included (Additional file 5).

Fifteen studies employed a qualitative study design; three employed a mixed-method study design, one involved a case-study and another adopted phenomenological approach. All studies used either in-depth interviews (*n* = 8) or focus group discussions (*n* = 8), or both (*n* = 4). Of the 20 studies, 15 evaluated both the barriers and facilitators of EBF practice, four evaluated barriers to EBF practice only and 1 assessed the facilitators of exclusive breastfeeding practice only.

### Barriers to the practice of exclusive breastfeeding (EBF) – thematic analysis

The thematic data analysis identified three themes, and eight sub-themes from 19 studies (Table 1) as barriers to EBF by the breastfeeding mothers.

**Table 1:** Major themes and sub-themes of barriers to EBF practice

Themes	Sub-themes	Codes	Studies
1.Maternal-infant factors	1.1 Maternal factors	1.1.1. Mother’s formal (employment) and informal work schedules	[26–33]
		1.1.2 Perceived breast milk insufficiency	[26–28, 31–38]
		1.1.3 Concerns of effects on mother’s appearance	[28, 31, 38, 39]
		1.1.4 Poor understanding/lack of awareness of EBF benefits	[32, 34, 35, 40]
		1.1.5 Being HIV positive or fear of transmitting HIV infection to the child	[31, 32, 34, 36]
		1.1.6 Schooling or resuming school or work	[29, 33, 36, 37, 41]
		1.1.7 Poor maternal nutrition	[29, 32–34, 41]
		1.1.8 Maternal perceived discomfort and embarrassment	[26, 28, 29, 31, 33, 39]
		1.1.9 Reluctance to breastfeed in public/disapproval of public breastfeeding	[29, 31, 33, 37]
		1.1.10 Refusal of mothers to breastfeed for several other reasons	[31–34, 41, 42]
	1.2 Infant factors	1.2.1 Crying baby	[31, 32, 37, 41, 42]
		1.2.2 Infant’s difficulty in latching or positioning or refusal to breastfeed	[31, 33, 37]
		1.2.3 Infant other issues	[30–32]
	1.3 Breast-related factors	1.3.1 Some breast conditions (cracked, painful or sore nipples)	[27, 28, 30, 32, 33, 42]
		1.3.2 Breast milk lightness and lousy odour	[39]
2. Support structures related factors	2.1 Family influence	2.1.1 Influence of husband	[26, 28, 29, 31, 33, 35, 37, 39]
		2.1.2 Influence of mother/mother-in-law/grandmother	[27, 29, 31, 33, 37–39, 41]
		2.1.3 Influence of other family members and important others	[27, 28, 31–37, 41, 43]
	2.2 Influence of health systems	2.2.1 Influence of advice or messages shared by HCWs	[28, 30, 33, 36, 37, 41]
		2.2.2 Inadequate breastfeeding education, counselling, and support by HCW	[28, 33, 38]
	2.3 Influence of workplace	2.3.1 Lack of workplace support	[28, 29, 40]
		2.3.2 Short and/or unpaid maternity leave	[28, 40, 41]
3. Influence of traditional and sociocultural beliefs	3.1 Use of herbal concoctions for medicinal purposes	3.1.1 Use of traditional herbal concoctions as medicine	[26, 32, 34, 39]
	3.2 Norms and beliefs	3.2.1 Traditional / cultural practices, myths, and misconceptions about EBF	[29–35, 39, 42–44]

### Maternal-infant factors

Several studies reported maternal, infant and child factors that served as barriers to the practice of exclusive breastfeeding. Eight studies [26–33] reported maternal employment and heavy workloads or schedules as barriers to exclusive breastfeeding. Mothers said that their tight work schedules interfered with the practice of exclusive breastfeeding as they revealed they could not carry the infant along when performing some work or errands. Mothers also reported that farming, gardening, cooking, and fetching water all limited their ability to breastfeed as they were exhausted and tired afterward and could not breastfeed their baby exclusively. Typical statements reported in the studies were as follows:

*“If I have much work [and] she wakes up, I can give her much formula so that she sleeps. So, it (introduction of other foods) was mainly to free me for more time for work”* ([28], pg. 4)

Perceived breast milk insufficiency was a critical barrier to EBF, as reported in 12 studies [26–28, 31–38]. Mothers were concerned about their breast-milk sufficiency and said that insufficient breast milk was a major hindrance to attaining optimum exclusive breastfeeding. This situation left mothers in a state of despair. One participant lamented:

*“I start to give my child cow milk at the age of 3 months because I believe that only my breast milk was not enough, and I was afraid my baby would get hungry”* ([35], pg. 10)

Four studies [28, 31, 38, 39] reported that mothers did not breastfeed their children exclusively because they associated exclusive breastfeeding with unwanted changes in physical appearance such as sagging breasts, weight gain or weight loss. Mothers, especially young ones, were more concerned about the perceived effect of EBF on their appearance as they feared they would not look good enough for men. Participants who were HIV-positive complained that they lost weight and felt unenergetic which they associated with frequent breastfeeding. One participant stated:

*“In our community, young girls like me (aged 18 – 25 years) do not like to breastfeed. They are afraid that the breasts will sag and their body shapes can change. My friends ask me: why are you breastfeeding? My child is four months now.”* ([39], pg. 4)

Four studies [32, 34, 35, 40] reported breastfeeding mothers demonstrated poor awareness and understanding of exclusive breastfeeding which affected their practice. These studies revealed that mothers did not understand what constitutes exclusive breastfeeding (i.e. what it involves and for how long it is recommended) and could not differentiate exclusive from partial and predominant breastfeeding, as demonstrated by the quote below:

*“A woman is obliged to breastfeed her baby until at least two years and it’s our culture. I gave all my children water, goat and cow milk mixed with water and sometimes biscuits soaked with milk. I didn’t give solid foods like bread or ‘enjera’ and I believe with my breast milk, this is the right way to raise my only 3-month-old baby.”* ([35], pg. 8)

Furthermore, widespread HIV and AIDS have affected breastfeeding practices, as reported in four studies [31, 32, 34, 36]. Mothers who were HIV-positive seek alternative ways of feeding their babies, thus discouraging them from practicing exclusive breastfeeding. Most mothers were fearful of breastfeeding their infant if they were HIV positive and did not want to infect their children.

*“Now, the coming of the disease called HIV makes breastfeeding difficult. To prevent transmission to a child you will be asked not to breastfeed. The breast may be full and painful, but you can’t breastfeed. It’s difficult, even if the baby is crying, you can’t give breast milk.”* ([31], pg. 38)

Furthermore, resuming school or work were indicated as barriers to EBF in five studies [29, 33, 36, 37, 41]. These mothers complained that breastfeeding was time-consuming and they faced difficulties integrating breastfeeding into their work/school schedule and chose bottle-feeding because it gave them more freedom to carry out their day-to-day activities. One participant stated:

*“Yes, I’m mixed feeding him because he eats formula when I’m not at home, during the day and then when I come back, he stops the bottle and breastfeeds.”* ([37], pg. 9)

Five studies [29, 32–34, 41] reported that poor maternal nutrition was a significant barrier to EBF with participants stating that infants are a product of what their mothers eat. Participants also pointed out that proper nutrition during breastfeeding is crucial to practicing EBF for a full 6 months, which was a challenge for some mothers as most were unable to maintain proper diets. One participant stated:

*“*. *.. .but [however] hungry, l would feel as if the baby is pulling me; as if the baby is suckling or draining my blood. .. because you will be feeling as if what the baby is sucking is no longer milk but my blood.”* ([32], pg. 41)

The practice of EBF causes much discomfort and sometimes embarrassment to breastfeeding mothers, as reported in six studies [26, 28, 29, 31, 33, 39].

*“You could sit in church, in the market or somewhere with people and your dress just becomes wet around the nipples. It’s very embarrassing especially if there are men there.”* ([33], pg. 4)

Social disapproval of breastfeeding in public and breastfeeding mothers’ reluctance to breastfeed their infants in places like schools, markets, churches, the workplace and public transport was reported in four studies [29, 31, 33, 37] as barriers to exclusive breastfeeding. Mothers also stated that they felt uncomfortable breastfeeding around men due to the perception of women’s breasts as sexual objects. This perception made mothers reluctant to breastfeed in public and influenced their choice to bottle-feed or give their infants water.

*“It’s a normal thing, but when you do it people stare at you like you’re doing something strange, especially men. Sometimes, some people will even ask you why you are breastfeeding in public.”* ([33], pg. 6)

Six studies [31–34, 41, 42] reported that some women refuse to breastfeed their infants for reasons ranging from personal views or circumstances, unplanned pregnancy and sickness, to the fear of falling sick and dying. Some mothers explained that they stopped EBF early and gave other foods to their children to get them accustomed to taking anything except breast milk in case they fell sick and died.

*“I just thought I could get sick, so I wanted my child to get used to eating other food, so if I die, he will be able to survive.”* ([42], pg. 5)

Factors relating to the infant which posed as barriers to EBF included crying associated with a hungry or unsatisfied baby, latching difficulties or refusal to breastfeed, teething, illness underweight and/or male infants. The mothers in five studies [31, 32, 37, 41, 42] complained of the persistent crying of their infants, especially as they grew older, which discouraged them from exclusive breastfeeding and led them to introduce water and/or other foods because they interpreted the crying as meaning the baby was still hungry. One participant stated:

*“When I breastfed, the baby will still be crying; then I make porridge which would be light [and] give [that to the] baby to drink, and the baby would stop crying and. .. starts playing.”* ([32], pg. 38)

Participants also reported that infant sickness prevents suckling, impairing mothers’ ability to exclusively breastfeed. One participant stated that some infants have weak jaws which impair their ability to suckle, leads to infant crying and prompts the mother to give other food. Sick babies lose interest in breastfeeding and can become irritated when the mother attempts to breastfeed. Mothers are then forced to try different foods to prevent the child from losing weight or getting sicker. In some cases, the child refused to latch onto the breast, causing the mother to introduce other foods.

*“No it’s different, a baby girl will breastfeed in a normal way, whereas a baby boy breastfeeds significantly. .. like a baby boy, if he breastfeeds from the mother, you see him crying then you know he is not getting enough, and then we give him porridge.”* ([32], pg. 35)

Breast conditions like swollen breasts, sore, cracked or painful nipples and breast milk’s light color and odor were also barriers to babies being exclusively breastfed reported in six studies [27, 28, 30, 32, 33, 42]. Mothers indicated that nipple pain, especially during the first 2 weeks of breastfeeding, was one factor that forced them to introduce alternative foods earlier than anticipated.

*“Assuming you have a boil or sore on the nipples, it becomes a serious challenge to breastfeed exclusively.”* ([27], pg. 10)

Other participants mentioned that breast milk has a foul odor which can be smelled by others, with some mothers hesitant to express breast milk due to its odor. Some breastfeeding mothers were also concerned about the quality of breast milk, stating that because the color is very light it cannot fill the child’s stomach, so they introduced other foods [39]. Participants stated:

*“I cannot practice EBF because breast milk is very light and the child won’t be full”* ([39], pg. 4)

### Support structures related factors

Another critical factor in the practice of EBF is support structures. Such structures can involve the family, the health system, or the workplace. Several studies have reported the varying influence of support structures as barriers to exclusive breastfeeding. Participants in eight studies [26, 28, 29, 31, 33, 35, 37, 39] stated that their husbands condemned EBF because of their insistence that infants should be given water. One participant stated:

*“The mere mention of the denial of water [when] exclusively breastfeeding. .. made my husband condemn the [practice].”* ([29], pg. 5)

Additionally, a lack of support from mothers, mothers-in-law and grandmothers was reported in eight studies [27, 29, 31, 33, 37–39, 41]. This lack of support made exclusive breastfeeding. Impossible as some mothers, mothers-in-law and grandmothers administered water or other foods to the baby without the breastfeeding mother’s consent or even threatened to leave the house if EBF was practiced.

*“When my mother visited, she told me not to adopt EBF practice. She said that if I want to give the baby only breast milk, she will pack her bags and leave because she cannot watch me punish the baby. I only obeyed my mother because I needed her care and assistance during the period”* ([29], pg. 5)

A further ten studies [27, 28, 31–33, 35–37, 41, 43] reported that other family members such as aunties and other relatives or close associates (co-tenants, nannies or house-girls) sometimes challenged mothers on the practice of exclusive breastfeeding.

The influence of health systems including advice or messages shared by Healthcare Workers (HCWs), inadequate support and poor EBF education among HCWs were also barriers to exclusive breastfeeding. Advice or messages shared by HCWs that were conflicting, inaccurate, outdated, or misleading, discouraged mothers from exclusive breastfeeding as reported in six studies [28, 30, 33, 36, 37, 41]. Participants also said that HCWs advised them to give water or infant formula immediately after delivery if breast milk flow was delayed, especially if the birth was a cesarean section or the baby started crying. Participants stated:

*“I had little challenges with some health workers too. It was only the doctor that was advising me positively. Some of the workers were like, don’t mind the doctor, if you must breastfeed your baby, it should be like three months, and that should be ok.”* ([30], pg. 8)

Inadequate support and poor exclusive breastfeeding education received from HCWs were reported as barriers to EBF in three studies [28, 33, 38]. Participants complained of receiving inadequate training, education and counselling from HCWs. Some even reported that health professionals offered the babies alternative feeds with or without the mother’s knowledge. One participant complained:

*“Nobody ever showed me how to breastfeed my baby. It was not as easy as I thought it was, but with time I learned. I think if a nurse could come and spend just like five or ten minutes with me talking about breastfeeding, it could help me to open up.”* ([33], pg. 8)

Inadequate workplace support was also reported in three studies [28, 29, 40] as barriers to EBF by working mothers. Participants complained that long hours of work did not allow them time to breastfeed adequately. Additionally, those who expressed breast milk at work lacked designated facilities, with most expressing breast milk in their offices, store, car, or worse still, in the toilet.

*“I can’t do this exclusive breastfeeding thing because, in school, my headmaster keeps shouting at me that it is taking away work time. He even asked me to [leave].*. *.the baby at home. .. I don’t want to lose my job.”* ([29], pg. 6)

Participants in three studies [28, 40, 41] stated that short and/or unpaid maternity leave meant they did not breastfeed their children exclusively. Mothers urged that the 3 months’ maternity leave be increased to enable them to practice EBF for the recommended 6 months, complaining:

*“Maternity leave is only three months, how can we practice EBF for six months?”* ([41], pg. 4)

### The influence of traditional and socio-cultural beliefs

Some sociocultural beliefs make the practice of EBF difficult as breastfeeding mothers often feel compelled to succumb to the dictates of their culture. The use herbal concoctions as medicines were reported by participants as barriers to exclusive breastfeeding. Mothers in four studies [26, 32, 34, 39] reported that participants gave gripe water and/or traditional medicines to infants to prevent or treat infantile distress. Such concoctions were usually acquired from, or advised by, other family members, neighbors, local mothers and healthcare workers.

Eleven studies [29–35, 39, 42–44] reported some traditional/cultural practices or EBF myths and misconceptions that posed barriers to exclusive breastfeeding. These practices included: circumstances that make the breast milk dirty/unclean (pregnancy and extra-marital affairs); giving the baby water; the effect of the baby burping on the breasts; fear of the evil eye; and other cultural norms and values.

*“The main barrier that I may highlight is cultural norms and values. You find that in this African setting when a woman gets married and lives with her in-laws, [when she gives birth], it is said that the baby belongs to the family of the husband to the extent that she is supposed to live according to the standards and norms of that particular family. If the mother-in-law believes that the babies are supposed to be given solid foods even at birth, even the pre-lacteal feeds might be gruel or something else; she will be forced to do that even when she doesn’t want [to], for marital security.”* ([32], pg. 44)

### Facilitators to the practice of exclusive breastfeeding

The thematic data analysis identified five themes and eight sub-themes from 16 studies as facilitators to the way of EBF by the breastfeeding mothers (Table 2).

**Table 2:** Facilitators to EBF practice: Codebook with the various identified themes, sub-themes, and studies

Themes	Sub-themes	Codes	Studies
1.Maternal-infant factors	1.1 Maternal factors	1.1.1 Mother’s personal opinion of EBF and knowledge of the benefits of EBF.	[29, 31, 32, 37, 39–41, 45]
		1.1.2 Mothers’ commitment and self-efficacy	[35–37, 40]
		1.1.3 Adequate maternal nutrition	[32, 34, 45]
	1.2 Infant factors	1.2.1 Better cognitive development	[26, 29, 30, 41, 45]
		1.2.2 Protection against infections	[29, 31, 32, 37, 39, 42]
2. Influence of support structures	2.1 Influence of family members	2.1.1 Influence of husband	[26, 29, 30, 32, 44]
		2.1.2 Influence of other family members	[26, 30, 32, 34, 36, 44]
	2.2 Workplace support	2.2.1 Flexible work time and longer maternity leave	[28, 41]
		2.2.2 Arrangement of nursery at the workplace	[40]
	2.3 Health system support	2.3.1 Influence of healthcare workers	[26, 28, 29, 36, 37, 41, 44]
3. Influence of traditional and sociocultural beliefs	3.1 Sociocultural beliefs	3.3.1 Breast milk is the only food for infants and a natural gift from God	[30, 39]
		3.3.2 Breastfeeding is a traditional practice	[29, 30]
		3.3.3 Belief that colostrum is good for the baby	[41, 45]
		3.3.4 Fear of discrimination	[28, 30, 38, 43, 45]
4. Influence on family finances	4.1 Resource constraints	4.1.1 Saves the cost of purchasing infant formula/hospital visits	[30, 31, 39, 41, 43]
5. Environmental factors	5.1 Poor sanitation and hygiene practices	5.1.1 Fear of infection due to inadequate preparation of food and water	[31]

### Maternal-infant factors

Participants identified some factors related to both the mothers and their infants which served as facilitators to exclusive breastfeeding. Maternal factors included: mothers’ opinions of EBF and breastfeeding education; mothers’ commitment and self-efficacy, and adequate maternal nutrition. Eight studies [29, 31, 32, 37, 39–41, 45] reported that mothers’ opinions of EBF and knowledge of the benefits of EBF encouraged them to practice EBF despite pressures from their husband, other family members and the community at large. Mothers stated that they enjoyed breastfeeding their children as it creates happiness and bonding between mother and the baby. Some mothers indicated that breastfeeding symbolized the meaning of motherhood. Their knowledge of breast milk’s benefits for their babies was also an important motivation to continue EBF irrespective of the challenges. One participant stated:

*“I enjoy breastfeeding my baby. When my baby is happy, I am happy too.”* ([39], pg. 3)

Mothers who successfully practiced EBF demonstrated strong self-efficacy and held to their own beliefs despite facing challenges as reported in four studies [36–38, 40]. Mothers explained that it took commitment and a firm resolve to exclusively breastfeed their babies because they had to deal with many obstacles from family members and in the workplace.

*“You know with breastfeeding; it’s just commitment. If you tell yourself that you want to do this, you will do it no matter what people might say. Even if there are stumbling blocks along the way, so I think it is all about committing yourself.”* ([37], pg. 10)

Three studies [32, 34, 45] reported the role adequate maternal nutrition played in facilitating exclusive breastfeeding. Mothers linked their ability to practice EBF to adequate feeding and fluid intake.

*“Adequate feeding and fluid intake, especially tea by a breastfeeding mother, will enable her breast to pump enough milk that will satisfy her baby.”* ([45], pg. 114)

Participants admitted that their motivation to EBF came from the fact that they wanted to have intelligent and healthy babies free from infections, especially HIV, cough and diarrhea, as reported by 11 studies [26, 29–32, 34, 37, 39, 41, 42, 45].

*“An exclusively breastfed baby hardly falls sick, grows rapidly, is very intelligent and above all is free from all infectious diseases.”* ([29], pg. 5)

### Support structure-related factors

Participants reported three support structure-related factors that facilitated exclusive breastfeeding. These structures were identified as: influences from family members, workplace support and healthcare services support. Seven studies reported that support from family members encouraged breastfeeding mothers to exclusive breastfeeding. Such support includes that received from husbands, mothers, mothers-in-law, other family members and nannies/house-helpers. Five studies [26, 29, 30, 32, 44] reported that mothers considered support from the husband as important to successful exclusive breastfeeding. This support could be economic through ensuring that mothers had enough food, and emotional through the spouse being faithful as unfaithfulness was reported as causing stress and worry for a breastfeeding mother, especially during the HIV pandemic.

*“To have one faithful partner so that you don’t have stress [and can] be emotionally and mentally stable and concentrate. I would say with my second baby, my husband [was] … unfaithful at that time. Men should refrain from unfaithfulness so that we can breastfeed well with our minds settled [and knowing] that we will not be infected by the virus that may end up infecting the baby as well.”* ([32], pg. 49)

Six studies [26, 30, 32, 34, 36, 44] reported that other family members (mothers, grandmothers, mothers-in-law, aunties, sisters) neighbors and the community all play essential roles in the promotion of exclusive breastfeeding. Mothers also received support which encouraged them to initiate and practice EBF and for childcare and household chores.

*“My mother-in-law encouraged me to breastfeed my children but also my mother, especially with my child that was delivered at home, she encouraged me to breastfeed because there was a time when I wanted to go back to work but she insisted that I first wait for the baby to grow. I was patient*” ([32], pg. 49)

Additionally, disclosure of HIV status by breastfeeding mothers was identified as a factor that helped them (the mothers) to get support from her family to exclusive breastfeeding [36]..

Three studies [28, 40, 41] reported that the availability/provision of a nursery at the workplace, flexible work time or longer maternity leave would encourage mothers to exclusive breastfeeding. Participants reported that the availability/provision of a nursery at the workplace for breastfeeding mothers would promote exclusive breastfeeding and keep mothers at work. Participants also stated that breastfeeding breaks and flexible working hours reduce maternal stress and enable a woman to breastfeed correctly. Participants that were formally employed said that extending maternity leave would encourage them to EBF; those in the informal sector mostly needed support from their partners or other family members. As stated by participants, such aid would allow mothers to stay at home and correctly practice breastfeeding.

*“. .. the workplace allowing for flexi-time will not just enable breastfeeding, but it will reduce the stress of the mother of being away from her baby the whole day, which is very good.”* ([28], pg. 4)

Another factor identified as a facilitator of EBF was the influence of healthcare workers. This influence involving informative messages and practical advice for positioning and attachment, was reported in seven studies [26, 28, 29, 36, 37, 41, 44]. Mothers admitted receiving assistance from health professionals on correct positioning and latching of the baby which encouraged them to continue exclusive breastfeeding. HCWs also played a vital role in guiding HIV-infected mothers on decision-making around infant feeding, with several mothers only deciding to breastfeed upon receiving HCWs’ advice.

*“When we were at the clinic, we started by learning about feeding the baby for six months; not giving him water but make [ing] him drink breast milk only.”* ([36], pg. 6)

### The influence of traditional and sociocultural beliefs

Participants reported some traditional social and cultural beliefs which served as facilitators to exclusive breastfeeding. Two studies [30, 39] reported that the belief that breast milk is the only food for infants and a natural gift from God encouraged mothers to exclusively breastfeed their children. These participants considered breastfeeding as a religious privilege as they were able to provide their infants with the “natural milk from God” and therefore, the best possible food that could be given to their babies.

The belief that breastfeeding is a traditional cultural practice encouraged participants to EBF, was reported in two studies [29, 30].

*“Breastfeeding is a traditional practice. There is even an Igbo axiom that says that any child who does not suck breast milk well will not have common sense and will not be intelligent.”* ([29], pg. 5)

Two studies [41, 45] reported that the belief that colostrum is good for the baby served as a facilitator to the initiation and continuation of exclusive breastfeeding. Mothers agreed that all infants should be given colostrum because it is good for the infant’s immunity.

The fear of HIV-related stigma or discrimination if a mother did not breastfeed encouraged the decision to EBF, was reported in five studies [28, 30, 38, 43, 45]. Participants stated they practiced EBF because they wanted to avoid the feeling of being judged by people for using infant formula rather than breastfeeding. Participants also expressed concern over their husbands’ reaction, and the fear of stigma and discrimination if they deviated from the usual practice of breastfeeding. One participant stated:

*“If I don’t breastfeed, people will start asking questions and may suspect that I have HIV and will never buy from me or even come near me again.”* ([45], pg. 114)

### Influence on family finances

Five studies [30, 31, 39, 41, 43] reported that financial constraints were an important factor in some mothers’ decision to exclusive breastfeeding. Exclusive breastfeeding has been linked to fewer cases of infant illness infant and hospital visitations. During hospital visits, families spend a lot of money. This cost encouraged mothers to EBF because they could not afford infant formula, clean water and were unable to maintain standard sanitary practices for preparing feeding bottles. Participants also reported that their families encouraged them to practice EBF because it was economical. Unemployed mothers considered exclusive breastfeeding a better financial option than formula feeding.

*“It helps the family economy as the child won’t be sick, no need of going to the hospital.”* ([39], pg. 4)

### Environmental factors

Some environmental factors, especially the fear of infection, were reported as facilitators to exclusive breastfeeding [31]. Mothers admitted that fear of disease due to inadequate food preparation, water or substances introduced while bottle-feeding encouraged them to exclusively breastfeed their children.

## Discussion

The World Health Organization (WHO) recommends EBF from birth up to the age of 6 months, but this practice or non-practice is determined by a number of factors such as: sociodemographic factors such as maternal age, education, employment, antenatal care practices and cultural and religious practices. Several studies have been undertaken to quantitatively identify these factors globally, with only a few conducted in sub-Saharan Africa. However, no systematic review has qualitatively identified these factors from the breastfeeding mothers’ viewpoint. To this end, we aimed to identify those factors that are facilitators or barriers to the practice of EBF from the perspective of breastfeeding mothers. Evidence from the included studies revealed that breastfeeding mothers confronted a number of factors such as inadequate support structures, and traditional and sociocultural beliefs that served as barriers to the practice of EBF, but maternal-infant factors were identified as the most significant barrier to exclusive breastfeeding. Mothers’ perception of insufficient breast milk was one maternal-infant factor which many reported led them to introduce other foods before the recommended 6 months duration. These mothers found themselves in a state of despair when they felt their breast milk was insufficient to satisfy their infant. This finding is consistent with the results of other studies undertaken in developing countries [21]. The findings of this review also revealed that maternal work schedules (both formal and informal income-generating activities outside the home) contributed to the non-practice of EBF, a situation exacerbated by the short period of maternity leave for employed mothers. This finding was also reported in another review conducted in developing countries [21]. Additionally, the lack of support received from family members and healthcare workers discouraged mothers from exclusively breastfeeding.

The findings of this study also reveal that maternal-infant factors and support structures are primary facilitators of EBF, with the influence of healthcare workers being the most significant motivator. Among HIV positive mothers, fear associated with mixed feeding and transmitting the infection to the infant discouraged mothers from exclusively breastfeeding their children. Mothers, especially HIV positive mothers, trust healthcare workers as their main source of advice on infant feeding. The promotion of EBF for 6 months by healthcare workers was the clear and dominant message communicated to mothers during counselling.

The strength of this review is that it includes mothers with children older than 6 months and HIV positive mothers, as there are some unique factors that impact on this population’s practice of exclusive breastfeeding. A similar study conducted in developing countries did not include HIV positive mothers and women with children older than 6 months of age [21]. This study also conducted a narrative investigation without emphasizing the qualitative aspect of the breastfeeding mothers’ perspective [21]. In contrast, our study involved a qualitative study of breastfeeding in sub-Saharan Africa which focused on the experiences of breastfeeding mothers as the major factor upon which the practice of EBF depends.

Exclusive breastfeeding is a critical public health issue. Consequently, identification of the factors that encourage or discourage breastfeeding mothers to exclusively breastfeeding are of great importance. All stakeholders, especially healthcare workers, must understand these factors so they can proffer solutions to the barriers, and strengthen the facilitators of exclusive breastfeeding. The factors identified from this study can be used to inform the development of educational interventions employed by health facilities during antenatal and/or postnatal counselling. This strategy would improve women’s knowledge, understanding, acceptance and practice of EBF, especially among HIV-positive mothers. Support from family members could also be improved by ensuring they also attend antenatal and postnatal clinics to develop a positive attitude towards exclusive breastfeeding. Additionally, healthcare workers should be regularly updated on current infant feeding practices to ensure they provide accurate information and effective support to mothers, especially HIV-positive mothers. All health systems should develop a standardized counselling guide using information synthesized from this review and other high-quality studies. This guide should be used during routine counselling of all women of childbearing age accessing health care facilities during antenatal and/or postnatal clinics, to improve the rates of exclusive breastfeeding.

This review has some limitations. It presents only primary research among breastfeeding mothers living in sub-Saharan Africa. There is the potential for missing some studies because only a few databases were searched, despite the search including studies from 1990 to 2019 to ensure that the timeframe was wide enough to include enough studies. A further limitation was the exclusion of studies not available in the English language.

Regardless of the limitations, the findings are one of the strengths of this review is that it contributes to the literature on exclusive breastfeeding with an emphasis on the barriers and facilitators from the perspective of breastfeeding mothers. Globally, systematic reviews on exclusive breastfeeding are generally quantitative, with the few qualitative studies lacking in-depth knowledge on factors key informants and breastfeeding mothers see as barriers or facilitators to exclusive breastfeeding. A few qualitative reviews have explored the factors that promote or discourage breastfeeding mothers from exclusively breastfeeding their children, but none have been conducted in sub-Saharan Africa. Additionally, this review employed an approach (CASP tool) that assessed the risk of bias and methodological quality of the included studies. The methodological quality of most of the review studies was of high to moderate quality with most demonstrating a low risk of bias when assessed. Conversely, when the evidence was assessed across the studies, most of the evidence indicated a moderate risk of bias.

## Conclusions

This qualitative systematic review focused on identifying the barriers and facilitators to the practice of EBF in sub-Saharan Africa. The review sought in-depth knowledge from the perspective of breastfeeding mothers. Three major barriers and facilitators to EBF, namely, maternal-infant factors, the influence of support structures and the influence of traditional and sociocultural factors, were identified. Interventions that address these factors, especially maternal-infant factors as the prominent theme, will be useful when educating mothers during antenatal and postnatal clinics to improve and optimize the practice of exclusive breastfeeding. Such interventions could significantly and ultimately improve maternal and child health outcomes.

## Supplementary Information

**Additional file 1.** Search strategy on barriers and facilitators to exclusive breastfeeding practice in sub-Saharan Africa.

**Additional file 2.** PRISMA flow diagram.

**Additional file 3.** Data extraction tool.

**Additional file 4.** Characteristics of included studies.

**Additional file 5.** Critical Appraisal Skill Programme quality assessment tool for qualitative studies.

**Additional file 6.** PICOS and risk of bias assessment of included studies.

**Additional file 7.** Critical Appraisal Skill Programme tool qualitative evidence profile of identified barriers and facilitators to exclusive breastfeeding.

## Data Availability

All data generated or analyzed during this study are contained within the manuscript.

## Abbreviations

AIDS: Acquired immunodeficiency syndrome
BFHI: Baby-friendly Hospital Initiative
CASP: Critical appraisal skills programme
EBF: Exclusive breastfeeding
HCW: Health care workers
HIV: Human immunodeficiency syndrome
PRISMA: Preferred reporting items for systematic reviews and meta-analysis
PROSPERO: International prospective register of systematic reviews
UNICEF: United Nations Children’s Fund
WHO: World Health Organization.
