# Supplementary material for: A systematic review of qualitative research on barriers and facilitators to exclusive breastfeeding practice in sub-Saharan African countries
Source: Int Breastfeed J. 2021 Jun 5;16:44. doi: 10.1186/s13006-021-00380-6 (PMC8178897; doi:10.1186/s13006-021-00380-6)
Supplement: Supplementary file 1 — Additional file 1. Search strategy on barriers and facilitators to exclusive breastfeeding practice in sub-Saharan Africa. [file 13006_2021_380_MOESM1_ESM.docx]

**Additional file 1** Search strategy for barriers and facilitators to exclusive breastfeeding practice in sub-Saharan Africa

**Search Date – 31 OCTOBER 2019**

1. Breast feeding [mh]
2. (Exclusiv* [tiab] OR alone [tiab] OR only [tiab]) AND (breast feed* [tiab] OR breastfeed* [tiab] OR breastfed [tiab] OR breast fed [tiab] OR breast milk [tiab] OR breastmilk [tiab])
3. Breast milk [tiab]
4. Breastmilk [tiab]
5. #1 OR #2 OR #3 OR #4
6. Africa South of the Sahara [mh]
7. “Africa South of the Sahara”[tiab]
8. Sub-Saharan Africa* [tiab]
9. Subsaharan Africa* [tiab]
10. Angola*[tiab] OR Benin*[tiab] OR Botswana[tiab] OR Batswana[tiab] OR Burkina Faso [tiab] OR Burkina Fasso [tiab] OR Bobo-Dioulasso[tiab] OR Burkinabé[tiab] OR Burundi[tiab] OR Burundian*[tiab]
11. Cape Verde [tiab] OR Cabo Verde [tiab] OR Cabo Verdean*[tiab]OR Cameroon*[tiab] OR Central African Republic[tiab] OR Central African[tiab] OR Chad[tiab] OR Chadian*[tiab] OR Comoros[tiab] OR Comorian*[tiab] OR Comoran*[tiab] OR Congo[tiab] OR Congolese[tiab] OR Zaire[tiab] OR Zairean[tiab]
12. Brazzaville[tiab] OR Cote d'Ivoire[tiab] OR Ivory Coast[tiab] OR Ivorian*[tiab] OR Djibouti[tiab] OR Djiboutian*[tiab] OR Equatorial Guinea [tiab] OR Equatorial Guinean*[tiab] OR Equatoguinean*[tiab] OR Eritrea*[tiab] OR Eswatini [tiab] OR Swaziland[tiab] OR Swazi[tiab] OR Swazis[tiab] OR Ethiopia*[tiab]
13. Gabon[tiab] OR Gabonese[tiab] OR Gabonaise[tiab] OR Gambia[tiab] OR Gambian*[tiab] OR Ghana[tiab] OR Ghanaian*[tiab] OR Guinea[tiab] OR Guinean*[tiab] OR Bissau[tiab] OR Bissau-Guinean*[tiab]
14. Kenya[tiab] OR Kenyan*[tiab] OR Lesotho[tiab] OR Basotho[tiab] OR Liberia[tiab] OR Liberian*[tiab] OR Madagascar[tiab] OR Malagasy[tiab] OR Malawi[tiab] OR Malawian*[tiab] OR Mali[tiab] OR Malian*[tiab] OR Mauritania[tiab] OR Mauritanian*[tiab] OR Mauritius[tiab] OR Mauritian*[tiab] OR Mozambique[tiab] OR Mozambican*[tiab]
15. Namibia[tiab] OR Namibian*[tiab] OR Niger[tiab] OR Nigerien*[tiab] OR Nigeria[tiab] OR Nigerian*[tiab] OR Rwanda[tiab] OR Rwandan*[tiab] OR Sao Tome[tiab] OR Sao Tomean*[tiab]
16. Senegal[tiab] OR Senegalese[tiab] OR Seychelles[tiab] OR Seychellois*[tiab] OR Sierra Leone[tiab] OR Sierra Leonean*[tiab] OR Somalia[tiab] OR Somali*[tiab] OR South Africa[tiab] OR South African*[tiab] OR South Sudan[tiab] OR South Sudanese[tiab]
17. Sudan[tiab] OR Sudanese[tiab] OR Tanzania[tiab] OR Tanzanian*[tiab] OR Togo[tiab] OR Togolese[tiab] OR Uganda[tiab] OR Ugandan*[tiab] OR Zambia[tiab] OR Zambian*[tiab] OR Zimbabwe[tiab] OR Zimbabwean*[tiab]
18. #6 OR #7 OR #8 OR #9 OR #10 OR #11 OR #12 OR #13 OR #14 OR #15 OR #16 OR #17
19. #5 AND #18
20. ("1990/01/01"[PDAT] : "2019/05/30"[PDAT])
21. English [lang]
22. #19 AND #20 AND #21
23. Animals [mh] NOT Humans [mesh: noexp]
24. #22 NOT #23
