# Supplementary material for: A systematic review of qualitative research on barriers and facilitators to exclusive breastfeeding practice in sub-Saharan African countries
Source: Int Breastfeed J. 2021 Jun 5;16:44. doi: 10.1186/s13006-021-00380-6 (PMC8178897; doi:10.1186/s13006-021-00380-6)
Supplement: Supplementary file 3 — Additional file 3. Data extraction tool. [file 13006_2021_380_MOESM3_ESM.docx]

**Additional file 3** Data Extraction Tool

| **Data** | **Information** |
| --- | --- |
| **Bibliographic information**  Serial No & no from the search strategy  Medline or Forward citation  Country  Year |  |
| **Study aims**  Barriers  Facilitators |  |
| **Study design:** |  |
| **Method(s):**  Qualitative  Mixed method  Ethnographic designs  Case study Mixed method  Ethnographic designs  Case study  Grounded theory  Phenomenological study |  |
| **Sample:** |  |
| Search strategy |  |
| Size |  |
| Inclusion/exclusion criteria and participant characteristics |  |
| Data collection methods |  |
| Data analysis techniques |  |
| Ethical considerations and issues |  |
| **Results:** |  |
| Themes |  |
| Quotes |  |
| Author interpretations or explanations; |  |
| Strengths and limitations |  |
| **Conclusion** |  |
