# Supplementary material for: A systematic review of qualitative research on barriers and facilitators to exclusive breastfeeding practice in sub-Saharan African countries
Source: Int Breastfeed J. 2021 Jun 5;16:44. doi: 10.1186/s13006-021-00380-6 (PMC8178897; doi:10.1186/s13006-021-00380-6)
Supplement: Supplementary file 4 — Additional file 4. Characteristics of included studies. [file 13006_2021_380_MOESM4_ESM.docx]

**Additional file 4** Characteristics of included studies

| **No** | **Bibliographic information** | **Country** | **Study aim and setting** | **Setting** | **Study design**  **(Methods, participant’s description, and data collection method)** | **Results**  **(Barriers and/or Facilitators)** | **Author’s conclusion** | **CASP Assessment Score and description (Question(s) with assessment of “No” and/or “Unclear”)** |
| --- | --- | --- | --- | --- | --- | --- | --- | --- |
| 1. | Matare *et al*., 2019  (26) | Tanzania | To explore the barriers and facilitators to EBF in rural Tanzania and to  assess parents' willingness and ability to try specific recommended EBF  practices plus strategies for men to support breast-feeding. | Rural | Case study  36 households with infants  <6 months  Interview & Focus group discussion | **Facilitators**  1. Counseling from Health Care Workers (HCWs)  2. Supportive fathers.  **Barriers**  1. Home management of perceived infantile distress  2. Women’s workloads  3. Lack of support from others  3. Perceived breast-milk insufficiency | Exclusive breastfeeding promotion needs to address concerns about  infantile distress, help parents develop effective soothing techniques while  avoiding non-prescribed medicines. | 9.5: High-quality paper  (Question 9: Few illustrative quotes; limited explanation of results). |
| 2. | Tampah-Naah *et al*., 2019  (27) | Ghana | To explore challenges to breastfeeding practices by considering  spatial, societal, and maternal characteristics in Ghana. | Urban | Qualitative study  20 mothers aged  15-49 years  Interview | **Barriers**  1. Household chores.  2. Challenges due to work schedules.  3. Family influences on exclusive breastfeeding.  4. Low breast milk production  5. Swollen breasts or sore nipples | Addressing the challenges of breastfeeding would require co-creation of supportive environments  between couples and significant others as well as tackling institutional barriers  that obstruct adequate breastfeeding among mothers. | 9.5: High-quality paper  (Question 6: Researcher and participant relationship not discussed which could result in bias). |
| 3. | Mgongo *et al*., 2019  (39)  Tanzania | Tanzania | To explore  social and cultural factors that might influence the practice of breastfeeding  and exclusive breastfeeding in the Kilimanjaro region | Urban | Qualitative design  78 mothers with infants  aged 0-12 months  Focus group discussions  . | **Facilitators**  1. EBF is good for the family economy and prevents child sickness  2. Breast milk (BM) is the only food for infants.  3. Breastfeeding creates happiness.  4. BM is the best gift from mother to child.  **Barriers**  1. Breast milk is very light.  2. Breastfeeding can affect a mother’s appearance.  3. Breast milk has a bad odor.  4. Getting help from other family members.  5. Breastfeeding is tiring.  6. Circumstances that make the breast milk unclean.  7. Traditional remedies for infant illnesses  8. Fear of Evil Eye.  9. Effects of child burping on the mother’s breast | Beliefs that promote the practice of breastfeeding  could be used to develop breastfeeding messages to  improve breastfeeding practices. There is also a need to address beliefs  that interfere with the practice of exclusive breastfeeding in this setting. | 9.5: High-quality paper  (Question 6: Researcher and participant relationship not discussed which could result in bias). |
| 4. | Tsegaye *et al*., 2019  (35) | Ethiopia | To measure the prevalence and  identify associated factors of exclusive breastfeeding (EBF) practice in Afar,  Ethiopia. | Urban | Mixed method design  10 group of mothers  Focus group discussion | **Barriers**  1. Poor understanding of EBF  2. Traditional beliefs, myths, and misconceptions about breastfeeding  3. Insufficient breast milk production  4. Lack of support from husband and family  5. Husbands as a source of income  6. Husbands sharing jobs | The prevalence of EBF did not meet the World Health Organization  recommendations. Factors related to infrastructure, service delivery, health  education packages, and traditional beliefs were associated with EBF practice. | 9: High-quality paper  (Question 8: There is a lack of an in-depth description of the process of analysis).  (Question 10: Generalization may not be possible because of limited participants). |
| 5. | Horwood *et al*., 2019  (36) | South Africa | To explore decision-making about infant feeding  practices among HIV-positive mothers. | Rural and urban | Qualitative method  11 HIV-positive women aged between  15 and 41 years  Interview | **Facilitators:**  1. The role of HWs in the provision of infant feeding advice;  2. Importance of mothers’ self-efficacy to resist pressures from family and community to change her feeding practice.  3. Disclosure of HIV status was a factor that helped mothers to get support from family  **Barriers**  1. Perceived insufficient milk  2. Pressure from family members  3. Out-dated / inaccurate messages shared by HCWs  4. Fear of HIV transmission  5. Working mothers resuming work | Health workers play a pivotal role in providing  infant feeding support to HIV infected mothers, but need regular updates to  ensure if advice is correct and appropriate. | 8: Moderate-quality paper  (Question 5: Limited number of participants).  (Question 6: Researcher and participant relationship not clearly discussed, potential bias, and influence).  (Question 9: Limited explanation of results).  (Question 10: Generalization may not be possible because of limited participants). |
| 6. | Mgongo *et al*., 2018  (41) | Tanzania | To explore the knowledge, attitudes, and practices of EBF among mothers  in Kilimanjaro region of northern Tanzania | Rural & Urban | Qualitative method  78 mothers of infants aged  0-12 months  Focus group discussion | **Facilitators**  1. Advice from HCW.  2. Breastfeeding education  3. Longer maternity leave and support  **Barriers**  1. Poor maternal nutrition  2. Resuming work  3. Crying baby linked to the perception of insufficient milk supply  4. Advice from health care providers  5. Advice from close relatives, mothers, and mother-in-law | The women need support from close relatives and  employers to successfully practice EBF. | 10: High-quality paper. |
| 7 | Wainaina *et al*., 2018  (28) | Kenya | To explores the experiences of middle-income  women to understand their attitudes and practices of EBF and to contribute  toward the Baby-Friendly Hospital Initiative (BFHI) and Baby-Friendly Community Initiative  (BFCI) programs in Kenya. | Peri-urban | Qualitative method  21 middle-income  women with child < 2 years  In-depth  Interviews and focus group discussions | **Barriers**  1. Lack of workplace Support  2. Lack of health Professional Support  3. Inadequate social support (spouses, relatives, community, house help/nannies)  4. Psychosocial influences on exclusive breastfeeding practice | Women experienced inadequate social,  healthcare, and workplace support and preferred online sites for information on  breastfeeding than healthcare professionals or mass media.  . | 8: Moderate-quality paper.  (Question 5: Limited number of participants).  (Question 6: Researcher and participant relationship not clearly discussed, potential bias, and influence).  (Question 9: Limited explanation of results).  (Question 10: Generalization may not be possible because of limited participants). |
| 8 | Lang'at *et al*., 2018  (43) | Kenya | To identify  barriers to optimal feeding among HIV-exposed infants 0-5 months of age attending  a mission hospital in Bomet County, Kenya. | Urban | Qualitative study  35 HIV mothers with infants 0-5 months of age.  Interview and focus group discussion | **Facilitator**  1. Fear of HIV-related stigma in the local community if the mother chose not to breastfeed.  **Barriers**  1. Resource constraints  3. Social pressure from the family members  4. Conflicting knowledge of current guidelines and recommendations on infant and young child feeding  5. HIV-related stigma in the local community | Health worker retraining in and reinforcement of WHO guidance on  feeding HIV exposed/infected infants will clarify misconceptions around feeding  HIV exposed/infected infants, though there remain social and economic barriers to  full implementation. | 8: Moderate-quality paper.  (Question 5: Limited number of participants).  (Question 6: Researcher and participant relationship not clearly discussed, potential bias, and influence).  (Question 9: Limited explanation of results).  (Question 10: Generalization may not be possible because of limited participants). |
| 9 | Okafor *et al*., 2018  (29) | Nigeria | To investigate factors that influence Exclusive Breastfeeding Practice among Nursing Mothers in  rural areas of Enugu State and its implications for Social Work Practice in  Nigeria. | Rural | Qualitative study  60 nursing mothers  Focus group discussions | **Facilitators**  1. Health workers influence  2. Influence of support networks  3. The belief that it benefits the child and mother  **Barriers**  1. Cultural influence  2. Influence of support networks  3. Maternal nutrition  4. Maternal discomfort  5. Working mothers | The professional support from social workers is very vital to achieving conformity to  EBF practice. | 9: High-quality paper.  (Question 8: There is a lack of an in-depth description of the process of analysis).  (Question 9: Illustrative quotes are few; limited explanation of results). |
| 10 | Jama *et al*., 2017  (37) | South Africa | To prospectively explore enablers or barriers to  success among mothers who planned to exclusively breastfeed their infants for the  first six months of life, in KwaZulu-Natal, South Africa. | Rural & urban | Qualitative study  22 working women, teenagers, and  HIV positive pregnant women  In-depth interview | **Facilitators**  1. HCWs influence  2. Self-efficacy and commitment  3. The belief that it protects the baby from HIV  4. Breast milk was sufficient for the baby  **Barriers**  1. Lack of support from HCWs.  2. Maternal-baby factors (work schedules, unsatisfied baby).  3. Pressure from family  4. Returning to work or school | HCWs are key players in providing infant feeding  information and support. Strategies to improve HCWs  competency in infant feeding counseling are needed. | 8: Moderate-quality paper.  (Question 5: Limited number of participants).  (Question 6: Researcher and participant relationship was not discussed, potential bias, and influence).  (Question 8: There is a lack of an in-depth description of the process of analysis).  (Question 10: Generalization may not be possible because of the limited number of participants). |
| 11 | Coetzee *et al*., 2017  (30) | Nigeria | To identify and delineate the barriers to and facilitators of adherence to EBF amongst HIV-infected and uninfected women in Nigeria. | Urban | Qualitative study  37 (25 HIV-infected and 12 HIV-uninfected) women  In-depth interviews | **Facilitators**  1. Financial security  2. Fear of stigma if the mother chooses not to breastfeed  3. EBF is a traditional practice  4. Breast milk is a natural gift from God.  6. Support structures  **Barriers**  1. Physical problems)  2. Baby teething  3. Employment  4. Cultural beliefs and traditions | Most women preferred EBF and offered it to their infants. However, more efforts are needed to improve support structures at home and at work to accommodate women who choose to do EBF. | 8.5: Moderate-quality paper.  (Question 5: Limited number of participants).  (Question 9: Illustrative quotes are few; explanation of results is limited).  (Question 10: Generalization may not be possible because of the limited number of participants). |
| 12 | Ukegbu *et al*., 2011  (45) | Nigeria | To identify the factors influencing breastfeeding pattern among nursing mothers  in Anambra State, Nigeria. | Rural & urban | Mixed method study  10 nursing mothers  Focus group discussion | **Facilitators**  1. The belief that colostrum is good.  2. The beliefs that EBF benefits to mother and child  3. Adequate maternal nutrition  4. Influence of husband and fear of discrimination. | EBF rate was low among the mothers and the  factors identified to influence its practice have important implications to  breastfeeding intervention programs. | 7.5: Moderate-quality paper.  (Question 5: Limited number of participants).  (Question 6: Researcher and participant relationship was not discussed, potential bias, and influence).  (Question 8: There is a lack of an in-depth description of the process of analysis).  (Question 9: Limited explanation of results).  (Question 10: Generalization may not be possible because of the limited number of participants). |
| 13 | Østergaard & Bula, 2010  (38) | Malawi | To explore patterns of EBF as well as which factors  motivate or hinder women to practice EBF | Urban & semi-urban | Qualitative study  21 HIV positive women  Observations and in-depth  individual interviews | **Facilitators**  1. The commitment of the mother to practice EBF  2. The fear of discrimination  **Barriers**  1. Perceived lack of milk  2. The belief that it affects mothers appearance  3. Lack of control of the feeding situation  4. Felt and enacted stigma  5. Poor counseling | Intention itself is not a sufficient determinant  of successful EBF unless several enabling factors come together.  Programs must be sensitive to  social expectations to mothers and involve mothers-in-law and fathers in  counseling of mothers who intend to practice EBF. | 8.5: High-quality paper.  (Question 5: Limited number of participants).  (Question 8: There is a lack of an in-depth description of the process of analysis).  (Question 10: Generalization may not be possible because of the limited number of participants). |
| 14 | Arts *et al*., 2010  (44) | Mozambique | To identify the knowledge,  beliefs, and practices around exclusive breastfeeding--specifically, those of  mothers, fathers, grandmothers, and nurses--and to identify the support networks | Rural & urban | Qualitative study  95 mothers of children younger than 2 years  Focus group discussion | **Facilitators**  1. Support network  2. Influence of HCWs (adequate support, advice)  **Barriers**  1. Traditional practices | Interventions to improve exclusive breastfeeding should target family and community members and include training of HCWs in counseling. | 8.5: Moderate-quality paper  (Question 6: Researcher and participant relationship was not discussed, potential bias, and influence).  (Question 8: There is a lack of an in-depth description of the process of analysis).  (Question 9: Limited explanation of results). |
| 15 | Fjeld *et al*., 2008  (42) | Zambia | To collect baseline  information about knowledge, attitudes, and practices relevant to infant feeding, FGD was chosen as the main data  collection tool | Rural & urban | Qualitative study  81 mothers  Focus group discussion | **Facilitators**  1. To prevent disease as food would cause diarrhea.  **Barriers**  1. Fear that the mother could fall sick and die leaving the child  2. Perception of unsatisfied child  3. General breastfeeding problems  4. Breast milk insufficiency  5. Bad/dirty milk | EBF is beneficial for child health and the  deep-rooted beliefs  that prohibit EBF need to be addressed in projects and campaigns promoting EBF | 9.5: High-quality paper  (Question 4: Possible bias in recruitment strategy linked to the recruitment procedure). |
| 16 | Otoo *et al*., 2009  (31) | Ghana | To elicit the perceived incentives and  barriers to exclusive breastfeeding in Ghana | Peri-urban | Qualitative study  35 mothers with a mean age of 27.5 years and had at least  one child < 4 months old  Focus group discussion | **Facilitators**  1. The belief that “Breast milk is best”  2. Maternal factors (work schedules).  3. Expensive formula feeding  4. Infant formula is not easily accessible, its preparation is tedious and time-consuming  5. Infant factors (unsatisfied baby)  6. Environmental factors (poor hygiene)  **Barriers**  1. Maternal factors (work schedules)  2. Infant factors (unsatisfied baby)  3. Social and cultural factors:  3. Lack of support structures  4. Breast milk insufficiency | Addressing  the concerns put forward by these participants can be used to enhance exclusive breastfeeding  promotion in this region | 8: Moderate-quality paper.  (Question 5: Limited number of participants).  (Question 8: There is a lack of an in-depth description of the process of analysis).  (Question 9: Limited explanation of results).  (Question 10: Generalization may not be possible because of the limited number of participants). |
| 17 | Kumsa & Moges, 2019  (40) | Ethiopia | To identify facilitative factors determining the prevalence of exclusive breastfeeding in South-western Oromia, Jimma, and Woliso towns | Rural and urban | Mixed method  135 mother-infant pairs  Interview | **Facilitators**  1. Personal and social Factors  2. Knowledge of the benefits of EBF  3. Workplace support  **Barriers**  1. Lack of awareness about the benefits of exclusive breastfeeding  2. Short maternity leave | Several personal and social facilitating factors that can contribute to exclusive breastfeeding and help to devise policy direction promoting EBF were identified. | 9.5: High-quality paper.  (Question 9: Limited result explanation; Illustrative quotes are few). |
| 18 | Nduna *et al*., 2011  (32) | Zimbabwe | To explore factors that influence breastfeeding decisions and practices based on mothers' own breastfeeding experiences | Rural | Phenomenological research design  10 mothers  In-depth interviews | **Facilitators**  1. Adequate maternal nutrition.  2. Influence of support structures.  4 Knowledge of the benefits of breast milk.  **Barriers**  1. Use of herbal remedies as medicines.  2. Inadequate maternal nutrition.  3. Male gender feeds a lot.  4. The practice of giving water.  5. Breast milk insufficiency.  6. Crying baby.  7. Breast conditions.  8. Traditional and cultural norms and belief systems.  10. Influence of support structures.  12*.* Peer advise and misinformation  13. The HIV epidemic | Access to breastfeeding information and counseling services, fall far too short to produce the desired infant and young child feeding indicators. Myths and misconceptions around breastfeeding still exist and they are compounded by cultural norms, belief systems, values and societal expectations which negatively interfere with mothers‟ breastfeeding decisions, choices and practices | 8.5: Moderate-quality paper.  (Question 5: Limited number of participants).  (Question 6: Researcher and participant relationship was not discussed, potential bias, and influence).  (Question 10: Generalization may not be possible because of the limited number of participants). |
| 19 | Nduna *et al*., 2015  (34) | Zimbabwe | To explore factors that enable and hinder exclusive breastfeeding in a rural district of Zimbabwe | Rural | Qualitative study  10 mothers  Interview | **Facilitators**  1. Adequate maternal nutrition.  2. Influence of support structures  **Barriers**  1. Poor understanding of EBF  2. The practice of giving water  3. Traditional beliefs, myths, and misconceptions around breastfeeding.  3. Insufficient breast milk production.  4. Inadequate maternal nutrition  5. Mixed messages regarding HIV and breastfeeding | Breastfeeding messages ought to be context-specific, targeting custodians of tradition and belief systems, including men and significant others, to create a supportive and enabling environment for mothers to exclusively breastfeed. | 8: Moderate-quality paper.  (Question 5: Limited number of participants).  (Question 6: Researcher and participant relationship was not discussed, potential bias, and influence).  (Question 9: Few illustrative quotes).  (Question 10: Generalization may not be possible because of the limited number of participants). |
| 20 | Ngongalah *et al*., 2018  (33) | Cameroon | To explore the perceptions of mothers, caregivers and key informants on infant feeding in Cameroon, and barriers to exclusive breastfeeding  Rural & urban |  | Qualitative study  31 Nursing mothers, grandmothers and health workers  Interview and Focus group discussion | **Barriers**  1. Mother-baby factors  2. Influence of support structures  3. Socio-cultural influence  4. Influence of HCWs | Developing effective strategies to increase exclusive breastfeeding rates requires that mothers’ needs be understood and that influencing factors be addressed. Supportive environments are also needed for mothers to breastfeed exclusively | 8.5: Moderate-quality paper.  (Question 5: Limited number of participants)  .  (Question 9: Few illustrative quotes; a limited number of participants).  (Question 10: Generalization may not be possible because of the limited number of participants). |
