# Supplementary material for: A systematic review of qualitative research on barriers and facilitators to exclusive breastfeeding practice in sub-Saharan African countries
Source: Int Breastfeed J. 2021 Jun 5;16:44. doi: 10.1186/s13006-021-00380-6 (PMC8178897; doi:10.1186/s13006-021-00380-6)
Supplement: Supplementary file 5 — Additional file 5. Critical Appraisal Skill Programme quality assessment tool for qualitative studies. [file 13006_2021_380_MOESM5_ESM.docx]

**Additional file 5** CASP Quality Assessment Tool for Qualitative Studies

| **CASP Quality Assessment Tool** | Yes | No | Unclear |
| --- | --- | --- | --- |
| Question 1: Was there a clear statement of the aims of the research? | 20 | 0 | 0 |
| Question 2: Is a qualitative methodology appropriate? | 20 | 0 | 0 |
| Question 3: Was the research design appropriate to address the aims of the research? | 20 | 0 | 0 |
| Question 4: Was the recruitment strategy appropriate to the aims of the research? | 19 | 0 | 1 |
| Question 5: Was the data collected in a way that addressed the research issue? | 6 | 0 | 14 |
| Question 6: Has the relationship between the researcher and participants been adequately considered? | 10 | 0 | 10 |
| Question 7: Have ethical issues been taken into consideration? | 19 | 0 | 1 |
| Question 8: Was the data analysis sufficiently rigorous? | 11 | 0 | 9 |
| Question 9: Is there a clear statement of findings? | 11 | 0 | 9 |
| Question 10: How valuable is the research? | 6 | 0 | 14 |
