# Supplementary material for: A systematic review of qualitative research on barriers and facilitators to exclusive breastfeeding practice in sub-Saharan African countries
Source: Int Breastfeed J. 2021 Jun 5;16:44. doi: 10.1186/s13006-021-00380-6 (PMC8178897; doi:10.1186/s13006-021-00380-6)
Supplement: Supplementary file 6 — Additional file 6. PICOS and risk of bias assessment of included studies. [file 13006_2021_380_MOESM6_ESM.docx]

**Additional file 6** PICOS and risk of bias of included studies

| **No** | **Bibliographic information** | **Participants** | **Outcome** | **Study design** | **CASP Assessment for the risk of bias** |
| --- | --- | --- | --- | --- | --- |
| 1. | Matare *et al*., 2019  (26) | 36 households with infants  <6 months | Barriers and facilitators | Case study | Low risk |
| 2. | Tampah-Naah *et al*., 2019  (27) | 20 mothers aged  15-49 years | Barriers | Qualitative study | Low risk |
| 3. | Mgongo *et al*., 2019  (39) | 78 mothers with infants  aged 0-12 months | Barriers and facilitators | Qualitative design  . | Low risk |
| 4. | Tsegaye *et al*., 2019  (35) | 10 group of mothers | Barriers | Mixed method design | Moderate risk |
| 5. | Horwood *et al*., 2019  (36) | 11 HIV-positive women aged between  15 and 41 years | Barriers and facilitators | Qualitative method | Low risk |
| 6. | Mgongo *et al*., 2018  (41) | 78 mothers of infants aged  0-12 months | Barriers | Qualitative method | Low risk |
| 7 | Wainaina *et al*., 2018  (28) | 21 middle-income  women with child < 2 years | Barriers | Qualitative method | Low risk |
| 8 | Lang'at *et al*., 2018  (43) | 35 HIV mothers with infants 0-5 months of age | Barriers | Qualitative study | Moderate risk |
| 9 | Okafor *et al*., 2018  (29) | 60 nursing mothers | Barriers | Qualitative study | Moderate risk |
| 10 | Jama *et al*., 2017  (37) | 22 working women, teenagers, and  HIV positive pregnant women | Barriers and facilitators f | Qualitative study | Low risk |
| 11 | Coetzee *et al*., 2017  (30) | 37 (25 HIV-infected and 12 HIV-uninfected) women | Barriers and facilitators | Qualitative study | Low risk |
| 12 | Ukegbu *et al*., 2011  (45) | 10 nursing mothers | Barriers and facilitators | Mixed method study | Moderate risk |
| 13 | Østergaard & Bula, 2010  (38) | 21 HIV positive women | Barriers and facilitators | Qualitative study | Moderate risk |
| 14 | Arts *et al*., 2010  (44) | 95 mothers of children younger than 2 years | Barriers and facilitators | Qualitative study | Moderate risk |
| 15 | Fjeld *et al*., 2008  (42) | 81 mothers | Barriers | Qualitative study | Moderate risk |
| 16 | Otoo *et al*., 2009  (31) | 35 mothers with a mean age of 27.5 years and had at least  one child < 4 months old | Barriers | Qualitative study | Low risk |
| 17 | Kumsa & Moges, 2019  (40) | 135 mother-infant pairs | Barriers and facilitators | Mixed method | Moderate risk |
| 18 | Nduna *et al*., 2011  (32) | 10 mothers | Barriers and facilitators | Phenomenological research design | Low risk |
| 19 | Nduna *et al*., 2015  (34) | 10 mothers | Barriers and facilitators | Qualitative study | Low risk |
| 20 | Ngongalah *et al*., 2018  (33) | 31 Nursing mothers | Barriers | Qualitative study | Low risk |
