# Supplementary material for: A systematic review of qualitative research on barriers and facilitators to exclusive breastfeeding practice in sub-Saharan African countries
Source: Int Breastfeed J. 2021 Jun 5;16:44. doi: 10.1186/s13006-021-00380-6 (PMC8178897; doi:10.1186/s13006-021-00380-6)
Supplement: Supplementary file 7 — Additional file 7. Critical Appraisal Skill Programme tool qualitative evidence profile of identified barriers and facilitators to exclusive breastfeeding. [file 13006_2021_380_MOESM7_ESM.docx]

**Additional file 7** CASP tool qualitative evidence profile of identified barriers and facilitators to exclusive breastfeeding

| Barriers to Exclusive Breastfeeding Practice | | | |
| --- | --- | --- | --- |
| Key Theme: Beliefs and Perceptions | | | |
| Finding No. | Review Finding | Studies Contributing to the Review Finding | Assessment of Risk of Bias Across Studies |
| 1. | Mother’s formal (employment) and informal work schedules | 1,2,7,9,11,16,18,20 | Moderate risk |
| 2. | Perceived breast milk insufficiency | 1,2,7 | Low risk |
| 3. | Concerns of effects on mother’s appearance | 7,16,13,3 | Moderate risk |
| 4. | Poor understanding/lack of awareness of EBF benefits | 18,19,4,17 | Moderate risk |
| 5 | Being HIV positive and/or fear of transmitting HIV infection to the child | 16,18,19,36 | Low risk |
| 6. | Schooling or resuming school or work | 9,20,5,10,6 | Moderate risk |
| 7. | Poor maternal nutrition | 9,18,20,19,6 | Moderate risk |
| 8. | Maternal perceived discomfort and embarrassment | 1,7,9,16,20,23 | Moderate risk |
| 9. | Reluctance to breastfeed in public/disapproval of public breastfeeding | 9,16,20,10 | Moderate risk |
| 10. | Refusal of mothers to breastfeed for several other reasons | 16,18,20,19,6,15 | Moderate risk |
| 11. | Crying baby | 11,18,10,6,15 | Moderate risk |
| 12. | Infant’s difficulty in latching or positioning or refusal to breastfeed | 16,20,10 | Low risk |
| 13. | Infant other issues | 11,16,18 | Low risk |
| 14. | Some breast conditions (cracked, painful or sore nipples) | 2,7,11,18,20,15 | Moderate risk |
| 15. | Breast milk lightness and bad odor | 3 | Low risk |
| 16. | Influence of husband | 1,7,9,16,20,4,10,3 | Moderate risk |
| 17. | Influence of mother/mother-in-law/grandmother | 2,9,16,20,10,13,3,6 | Moderate risk |
| 18. | Influence of other family members and important others | 2,7,16,18,20,4,5,10,6,8 | Moderate risk |
| 19. | Influence of advice or messages shared by HCWs | 7,5,10,6 | Low risk |
| 20. | Inadequate breastfeeding education, counseling, and support by HCW | 7,20,13 | Moderate risk |
| 21. | Lack of workplace support | 7,9,17 | Moderate risk |
| 22. | Short and/or unpaid maternity leave | 7,17 | Moderate risk |
| 23. | Use of traditional herbal concoctions as medicine | 1,18,19,3 | Low risk |
| 24. | Traditional / cultural practices, myths, and misconceptions about EBF | 9,11,16,18,20,19,4,3,15,8,14 | Moderate risk |
| Facilitators to Exclusive Breastfeeding Practice | | | |
| Key Theme: Beliefs and Perceptions | | | |
| Finding No. | Review Finding | Studies Contributing to the Review Finding | Assessment of Risk of Bias Across Studies |
| 1. | Mother's personal opinion of EBF and knowledge of the benefits of EBF. | 9,18,16,10,3,17,6,12 | Moderate risk |
| 2. | Mothers’ commitment and self-efficacy | 5,10,13,17 | Moderate risk |
| 3. | Adequate maternal nutrition | 18,19,12 | Moderate risk |
| 4. | Better cognitive development | 1,9,11,6,12 | Moderate risk |
| 5 | Protection against infections | 9,16,18,10,3,15 | Moderate risk |
| 6. | Influence of husband | 1,9,11,18,14 | Moderate risk |
| 7. | Influence of other family members | 1,11,18,19,5,44 | Moderate risk |
| 8. | Flexible work time and longer maternity leave | 7,6 | Low risk |
| 9. | Arrangement of nursery at the workplace | 17 | Moderate risk |
| 10. | Influence of health care workers | 1,7,9,5,10,6,14 | Moderate risk |
| 11. | Breast milk is the only food for infants and a natural gift from God | 11,3 | Low risk |
| 12. | Breastfeeding is a traditional practice | 9,11 | Moderate risk |
| 13. | The belief that colostrum is good for the baby | 6,12 | Moderate risk |
| 14. | Fear of discrimination | 7,11,13,8,12 | Moderate risk |
| 15. | Saves the cost of purchasing infant formula/hospital visits | 11,16,3,6,8 | Moderate risk |
| 16. | Fear of infection due to inadequate preparation of food and water | 16 | Low risk |
